# Construction of hexabenzocoronene-based chiral nanographenes

**DOI:** 10.3762/bjoc.19.54

**Published:** 2023-05-30

**Authors:** Ranran Li, Di Wang, Shengtao Li, Peng An

**Affiliations:** 1 School of Chemical Science and Technology, Yunnan University, Kunming 650500, P. R. China

**Keywords:** chiral nanographene, helicene, racemization barrier, Scholl reaction, single-crystal X-ray diffractometry

## Abstract

The past decade witnessed remarkable success in synthetic molecular nanographenes. Encouraged by the widespread application of chiral nanomaterials, the design, and construction of chiral nanographenes is a hot topic recently. As a classic nanographene unit, hexa-*peri*-hexabenzocoronene generally serves as the building block for nanographene synthesis. This review summarizes the representative examples of hexa-*peri*-hexabenzocoronene-based chiral nanographenes.

## Introduction

Graphene, an allotrope of carbon, has captured widespread attention since it was first experimentally demonstrated as a monolayer of carbon atoms [1]. Graphene and graphene-based materials are consisted of sp^2^ carbons and have shown a variety of outstanding applications, particularly in electronic devices as conductor materials with a zero band gap [2–3]. Compared to infinite graphene, nanoscale graphene fragments exhibit size-dependent non-zero bandgaps, which significantly enhanced their semiconducting character [4–5]. Hence, the preparation of graphene segments, so-called nanographenes (NGs), has received much attention. In materials science, graphene fragments have been obtained by “cutting” graphene materials through specific chemical methods, which leads to non-homogeneous NGs [6]. To have structurally well-defined NGs, the chemical synthetic approach offers an alternative and superior access. As a result, a variety of synthetic molecular NGs have been prepared through a bottom-up approach in recent years [7–9]. These newly designed NGs with tailor-made sizes and structures not only show planar conformation as fragments of graphene, but also evolved with a diversity of other shapes, including bowls [10], saddles [11–13], helixes [14–15], belts [16], rings [17], and their hybrid conformations such as helical bilayers [18], and nanoribbons [19]. Meanwhile, to modulate the properties of synthetic hydrocarbon NGs, a variety of heteroatom-doped NGs have been constructed [8].

Chirality is a fundamental phenomenon in nature and plays an important role in science, especially in living systems. The chirality in chemistry refers to the molecules which cannot be superimposed on their own mirror image. Nanomaterials with chiroptical properties have shown potential applications in different fields such as optical devices, polarization-based information encryption, circularly polarized luminescence (CPL) lasers, or biosensing [20–24]. Currently, there is a great interest in the introduction of chirality into large conjugated polyaromatics to obtain chiral NGs with chiroptical properties. Among these chiral NGs, helicenes represent the dominant chiral compounds by virtue of their inherent helical chirality [25–28]. Meanwhile, the introduction of strain into polyaromatic systems to generate Gaussian curvature or twistedness offers an alternative approach to forming chiral NGs [14].

**Scheme 1:**
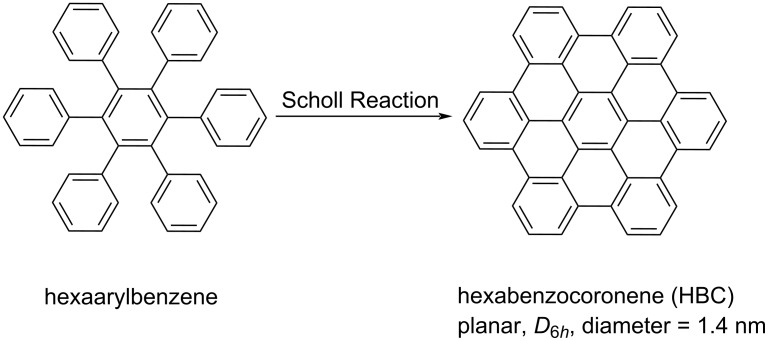
Construction of HBC by Scholl reaction from hexaphenylbenzene.

As a classic NG unit, hexa-*peri*-hexabenzocoronene (*p*-HBC), a *D*_6_*_h_*-symmetry, planar π-scaffold (Scheme 1), and its derivatives have attracted increasing attention due to their specific optoelectronic properties [5,29]. HBC thus generally serves as a reference structure or building block to construct chiral NGs which are currently receiving a lot of interest in carbon-based materials science. This review will mainly focus on representative examples of HBC-based chiral molecular NGs. All the examples in the context can be treated either as HBC-like monomers, or HBC-based dimers, trimers, tetramers, or oligomers.

The synthesis of HBC is shown in Scheme 1. Pioneered by Scholl [30] and Clar [31] and further developed by Müllen [29], HBC was simply constructed through the Scholl-type oxidative intramolecular cyclodehydrogenation of hexaphenylbenzene. Accordingly, in the current cases in this review, the HBC-based chiral NGs were all established through oxidative cyclodehydrogenation by employing different oligophenylene precursors.

## Review

### *seco*-HBC-based chiral NGs

**Scheme 2:**
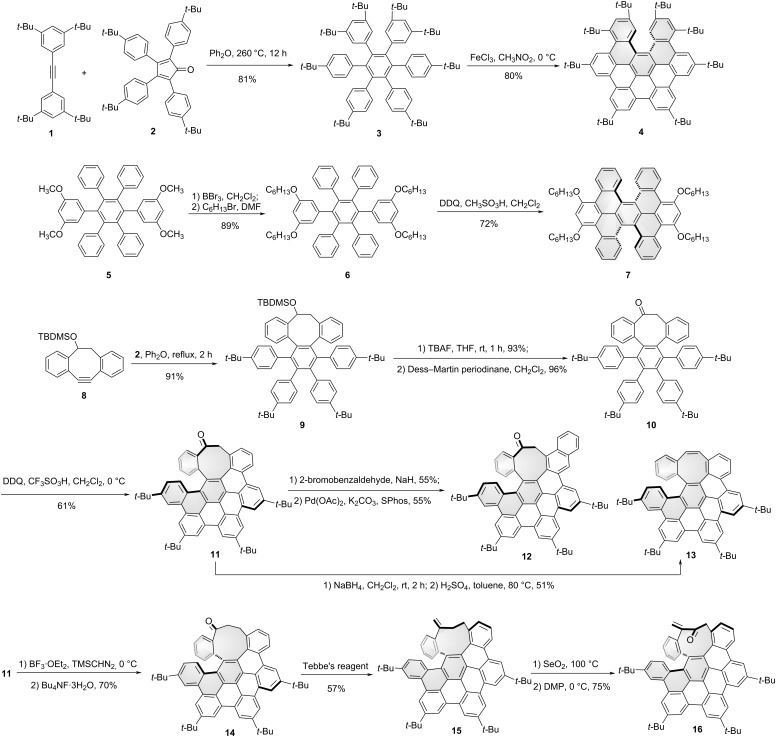
Synthesis of *seco*-HBC-based chiral nanographenes.

In the HBC synthesis, a small fraction of a partially cyclodehydrogenated intermediate was indeed isolated during the conversion of hexaphenylbenzene to HBC [32]. Conceptually, the partially ring-closed intermediate by cutting one C–C bond of HBC can be treated as *seco*-HBC, which gives a helical chiral structure. Based on this incomplete cyclizing strategy, types of *seco*-HBC-based chiral NGs were synthesized. Shown in Scheme 2 are the [5]helicenes that were synthesized from the corresponding hexaarylbenzenes as incompletely cyclized products during the Scholl reaction. To prevent planarization during the final oxidative cyclodehydrogenation reaction step, Jux and co-workers synthesized hexaarylbenzene precursor **3** with sterically demanding *tert*-butyl groups through a standard [4 + 2] Diels–Alder reaction of tetra-*tert*-butyltolane **1** and tetracyclone **2** in an 81% yield. The Scholl reaction of compound **3** in the presence of FeCl_3_ and nitromethane led to the [5]helicene containing, *seco*-HBC-based chiral NG **4** in an 80% yield [33]. Miao and co-workers reported a twisted chiral NG **7** by a partially cyclized Scholl reaction. By treating compound **6** in 2,3-dichloro-5,6-dicyano-*p*-benzoquinone (DDQ) and methanesulfonic acid in dichloromethane, the helical structure **7** was obtained in a 72% yield [34]. The possible reason for this incomplete cyclization is the electronic effect of the alkoxy groups. Meanwhile, the methoxy version was also synthesized from precursor **5**. The helical conformation was confirmed by the single-crystal X-ray crystallography of its methoxy-substituted analogue.

Another method to obtain the incompletely cyclized *seco*-HBC is the introduction of a non-hexagonal ring into the precursors of HBC. Due to the large ring strain, the precursors are not prone to form planar structures through the oxidative cyclodehydrogenation reaction. Campaña, Morcillo, and co-workers synthesized a series of *seco*-HBC-based chiral NGs by introducing an octagon or a nonagon ring into the HBC skeleton (Scheme 2). The octagon-containing hexaphenylbenzene **9** was prepared from dibenzocyclooctyne **8** and tetracyclone **2** in a 91% yield. After a subsequent sequence of deprotection and oxidation, ketone **10** was obtained. Through the oxidative cyclodehydrogenation reaction of **10** in the presence of DDQ and trifluoromethanesulfonic acid (TfOH), a saddle-helix hybrid nanographene **11**, bearing an octagon-containing carbo[5]helicene was isolated in a 61% yield, which can be further transformed into other chiral hydrocarbon analogues **12** and **13** [35]. Chiral resolution of these helicenes was accomplished by chiral high-performance liquid chromatography (HPLC) and the isomerization barriers were determined for the enantiopure helicenes. For compound **12**, the racemization barrier was 24.8 kcal mol^−1^ at 298 K, which provides a racemization half-life (*t*_1/2_) of 24.4 h. For compounds **11** and **13**, neither racemization nor decomposition was observed by chiral HPLC after heating hexadecane solutions of each compound at 200 °C for 5 h, which gives a lower limit to the barrier of racemization of Δ*G* > 38.3 kcal mol^−1^ at 473 K. The circularly polarized luminescence (CPL) of these structures were also evaluated with the luminescence dissymmetry factors (*g*_lum_) values of 4 × 10^−4^ for compound **11** and 7 × 10^−4^ for compound **12**. Meanwhile, Campaña, Morcillo, and co-workers converted the eight-membered ring **11** to nonagon-containing carbohelicene **14** through a single ring expansion reaction by treating **11** with TMSCHN_2_ (Scheme 2). By reacting with Tebbe’s reagent, compound **14** could be converted to all-carbon analogue **15**. And the derivative **16** was also synthesized by allylic oxidation of compound **15** using selenium dioxide. As helical chiral NGs, helicene **14** and its derivatives **15** and **16** showed highly distorted helical conformation and also exhibited a relatively high isomerization barrier (over 28.9 kcal/mol determined at 90 °C) [36].

**Scheme 3:**
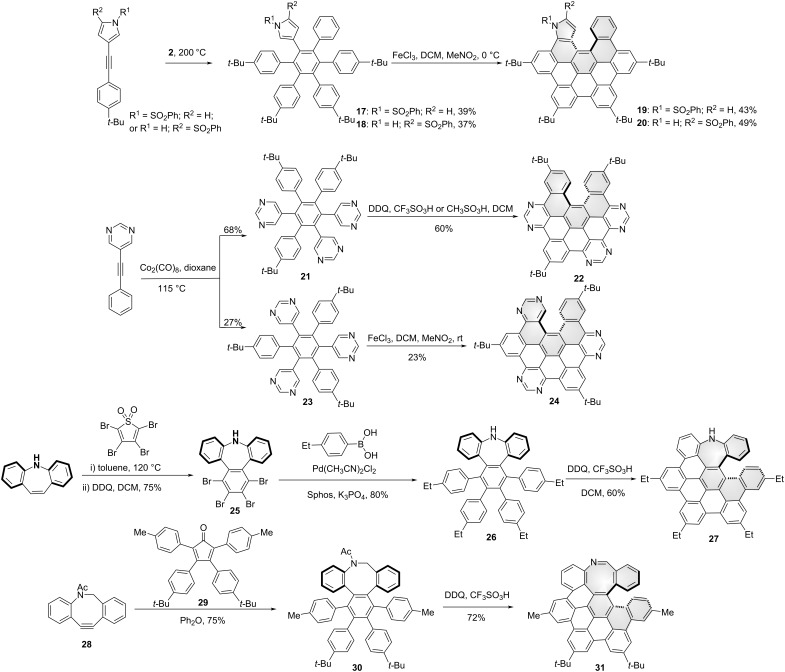
Synthesis of nitrogen-doped, seco-HBC-based chiral nanographenes.

It is now well-documented that the incorporation of heteroatoms into the graphene materials or NG molecules can effectively tune their electronic and optical properties. Examples in Scheme 3 show the nitrogen-doped, *seco*-HBC-based chiral NGs. By introducing a pyrrole ring, Jux and co-workers reported pyrrole-containing helical NGs **19** and **20**. The precursors **17** and **18** were synthesized from pyrrole-containing alkynes and tetracyclone **2** through a typical Diels–Alder reaction. The pair of enantiomers of these aza-[5]helicenes was confirmed by the X-ray crystal structure of racemic structure **13** [37]. Draper’s group reported the pyrimidine-containing N-doped NGs (Scheme 3). Tri-pyrimidine precursors **21** and **23** were obtained by trimerization of 5-(phenylethynyl)pyrimidine in the presence of Co_2_(CO)_8_. Upon treatment of the tri-pyrimidine containing hexarylbenzene compounds **21** or **23** under Scholl reaction conditions (DDQ, H^+^; or FeCl_3_, CH_3_NO_2_), the aza-[5]helicenes **22** and **24** were obtained respectively with 60% and 23% yields [38]. It was noted that with the installation of two adjacent pyrimidines in this hexarylbenzene precursor, a fully cyclized planar NG was formed toward Scholl reaction [39]. Recently, An and co-workers reported a helical, azepine-containing NG **27** by the introduction of an NH in the *seco*-HBC framwork (Scheme 3). Single-crystal X-ray diffractometry revealed the mixed *P*/*M* enantiomers. Taking advantage of the grafting NH functional group, the optical resolution of this helical aza-NG was achieved by introducing a chiral auxiliary reagent at the nitrogen site [40], and the racemization barrier of one enantiomer was measured as 26.2 kcal/mol by monitoring the changes of CD spectra at 60–80 °C. The synthesis started with the Diels−Alder reaction of 5*H*-dibenzo[*b*,*f*]azepine and tetrabromothiophene-*S*,*S*-dioxide, followed by oxidative aromatization in the presence DDQ to afford compound **25** in an overall 75% yield. Suzuki−Miyaura cross-coupling reaction of compound **25** with (4-ethylphenyl)boronic acid in the presence of Pd(CH_3_CN)_2_Cl_2_, SPhos, and K_3_PO_4_ then furnished the target hexaphenylbenzene **26** in an 80% yield. Compound **26** was treated with excess DDQ (10.0 equiv) in the presence of triflic acid to furnish the fourfold cyclization product **27** in a 60% yield. Notably, no fivefold cyclized product was detected. Meanwhile, by introducing an aza-eight-membered ring in the hexarylbenzene derivative **30**, An and co-workers also reported an azocine-embedded, [5]helicene containing NG **31** [41]. The precursor **30** was synthesized by Diels–Alder reaction of aza-alkyne **28** and tetracyclone **29**. By treating compound **30** under Scholl reaction conditions, the helical structure **31** was obtained through oxidative cyclodehydrogenation and imine formation in a 72% yield. It was noted that no other regioisomer was detected for the asymmetrical precursor **30**. The pair of enantiomers was confirmed by the racemic structure's single-crystal X-ray diffractometry and separated by chiral HPLC, and no racemization was observed by heating the enantiomer for 1 hour at 120 °C.

**Scheme 4:**
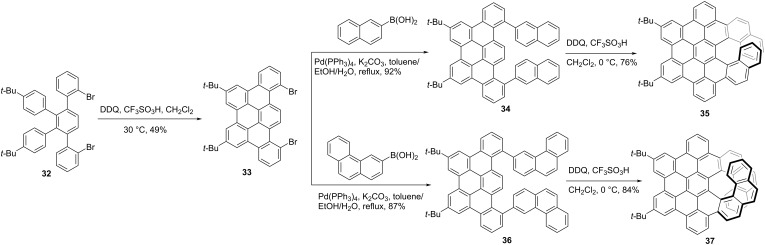
Synthesis of π-extended [7]- and [9]helicene containing chiral nanographenes.

Except for [5]helicene, Narita and co-workers also reported [7]- or [9]helicene-containing chiral NGs [42]. As shown in Scheme 4, dibromo-functionalized 1,2,3,4-tetraphenylbenzene **32** was treated with DDQ and TfOH to produce dibromo **33** as the prefused building block in a 49% yield. Then naphthalene and phenanthrene residues were introduced into compound **33** through Suzuki coupling reaction, the corresponding precursors **34** and **36** were obtained respectively in high yields. The final cyclodehydrogenation using DDQ and TfOH proceeded regioselectively at 0 °C, affording the desired π-extended [7]helicene **35** and [9]helicene **37** in 76 and 84% yields, respectively. Single-crystal structures of racemic **35** and **37** revealed the *P*/*M* enantiomers repectively, and the *P*/*M* racemization barriers of **35** and **37** were determined as 42.4 and 41.6 kcal/mol, respectively by DFT calculation. The enantiopure isomers of the π-extended helicenes were evaluated as excellent circularly polarized luminescence (CPL) emitters with a *g*_lum_ of 7.44 × 10^−3^ for **37**, which is around 10-fold higher than **35**.

### Chiral “HBC-dimers” and “HBC trimers”

**Scheme 5:**
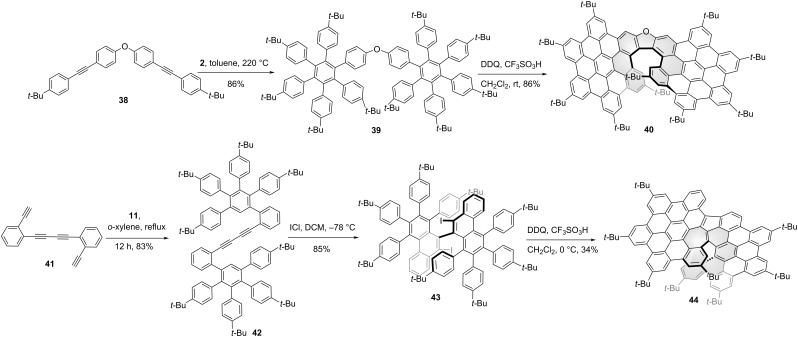
Synthesis of “HBC-dimer”-based chiral nanographenes.

The combination of two or more HBCs or HBC-like units into one ring system is a straightforward way to generate HBC-based chiral NGs. In general, different HBC units were fused together to form the final chiral structures by connecting with a linker or sharing the same phenyl ring. As shown in Scheme 5, Jux and co-workers synthesized the NG precursor **39** by heating dialkyne **38** and tetracyclone **2**, which led to two hexarylbenzene units connected by an oxygen linker. Upon treatment of **39** under Scholl reaction conditions (DDQ, TfOH), an oxa-[7]helicene containing chiral NG **40** was obtained in high yield [43]. Meanwhile, they enlarged this [7]helicene family by introducing different linkers such as nitrogen, sulfur, sulfone, ketone, methylene, and derivatives of ketone [44]. Due to the varied nature of the different linkers, the photophysical and semiconductor properties can be effectively tuned. Feng and co-workers reported a helical NG **44** containing [6]helicene structure and an azulene unit (Scheme 5). Through a two-fold Diels–Alder cycloaddition from 1,4-bis(2-ethynylphenyl)buta-1,3-diyne (**41**) and tetracyclone **11**, alkyne **42** was obtained in an 83% yield. Then unique diiodide precursor **43** was obtained by ICl-mediated benzannulation of diacetylene **42** in an 85% yield. The final NG **44** formed by treating compound **43** with DDQ/TfOH at 0 °C and the overall structure exhibits interesting photophysical and antiaromatic properties [45]. During the final Scholl reaction, an monoiodide structure **101** was also isolated (Scheme 11).

**Scheme 6:**
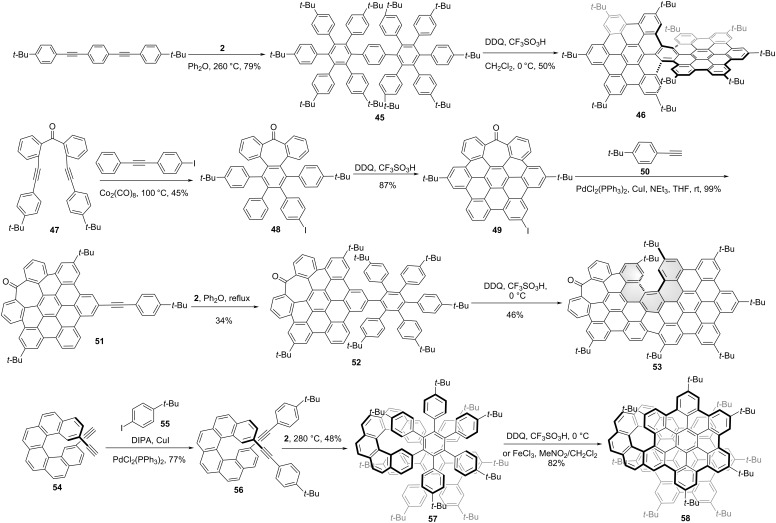
Synthesis of “HBC-dimer”-based chiral nanographenes.

In the pursuit of HBC-tetramer-based supertwistacene (compound **115** in Scheme 12), Wang and co-workers synthesized a chiral HBC-dimer **46** [46], in which two HBC units structurally shared one benzene ring (Scheme 6). 1,4-Bis((4-(*tert*-butyl)phenyl)ethynyl)benzene reacted with tetracyclone **2** through a two-fold Diels–Alder cycloaddition to afford compound **45** in a 79% yield. Due to the steric hindrance from two bulky *tert*-butyl groups on the benzene rings in the adjacent hexaphenylbenzene monomers in precursor **45**, two [5]helicenes were formed in the oxidative cyclodehydrognation reaction, which gave compound **46** as the *P*,*P*, and *M*,*M* enantiomers. The isomerization barrier was determined as 44.7 kcal/mol at 260 °C by HPLC analysis. Meanwhile, Campaña and co-workers reported a similar chiral, tropone-containing HBC-dimer **53** [47]. Ketone **47** was transformed to compound **48** by reacting with 1-iodo-4-(phenylethynyl)benzene through Co-catalyzed cyclotrimerization in a 45% yield. Then monoiodide NG **49** was obtained through oxidative cyclodehydronation in a high yield. From the heptagon-containing NG **49**, Sonogashira coupling with *p*-*tert-*butylphenylacetylene (**50**) afforded **51** in a quantitative yield. Subsequent Diels–Alder reaction with cyclopentadienone **2** gave polyphenylene **52** in a 34% yield. Unlike the double helical structure of NG **48**, the lack of the bulky *tert*-butyl group on one side in precursor **52**, only one [5]helicene was generated in the Scholl reaction during the formation of the final structure **53**. The Gibbs activation energy of enantiomer **53** for the racemization process was determined as 33.0 kcal mol^−1^ at 298 K. The CPL spectra of *M*-**53** and *P*-**53** showed an emission maximum at 560 nm with *g*_lum_ value of 2.3 × 10^−4^. Instead of helicene formation in the final Scholl-type ring formation step, Martín and co-workers synthesized a helical bilayer NG by using helicene in the initial step as the linker to fuse two HBC units [48]. As shown in Scheme 6, starting from the helical alkyne **54**, Sonogashira coupling with 4-*tert*-butyliodobenzene (**55**) afforded structure **56** in a 77% yield. Subsequent Diels–Alder reaction with cyclopentadienone **2** generated compound **57**. The final Scholl reaction of **57** afforded helical NG **58** in high yield. This folded NG **58** is composed of two HBC layers fused to a [10]helicene with an interlayer distance of 3.6 Å as determined by X-ray crystallography. Since the helicene was initially introduced as a linker, an optically pure chiral NG synthesis was accomplished using the same synthetic route by starting with an enantiopure helicene.

**Scheme 7:**
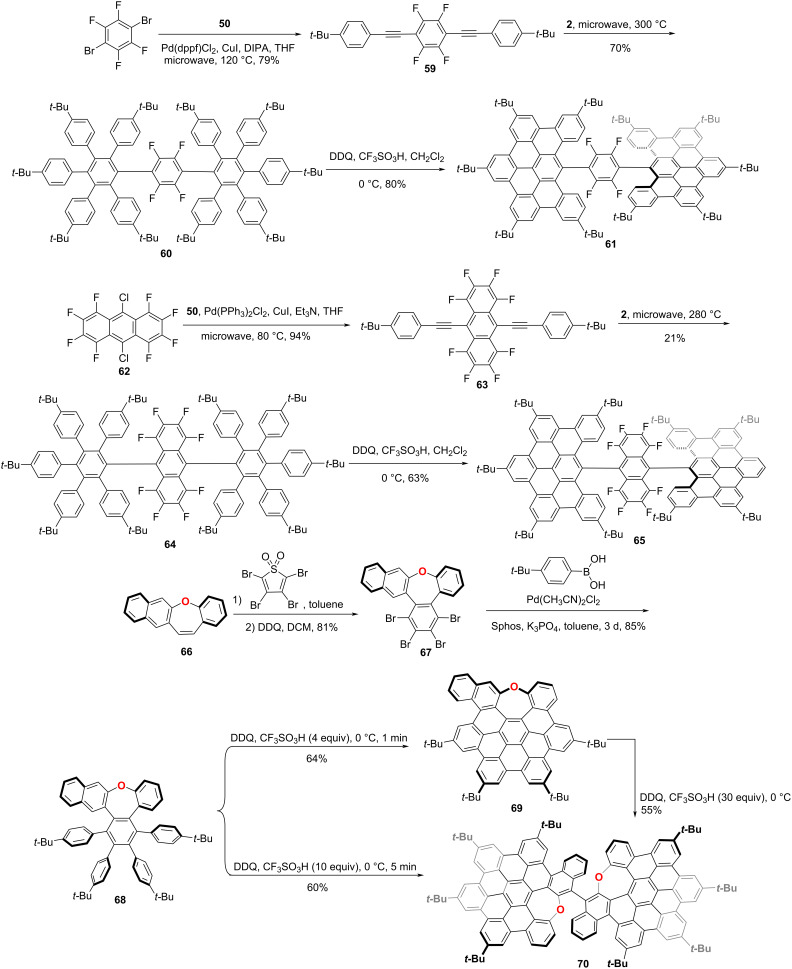
Synthesis of axis-based chiral nanographenes.

Except for the helicene-based chirality, chirality stemming from a chiral axis is relatively rare in designing chiral NGs. Notably, Martín and co-workers reported a new family of chiral NGs, which constituted by two orthogonal dibenzo[*fg*,*ij*]phenanthro-[9,10,1,2,3-*pqrst*]pentaphene (DBPP) units covalently connected through tetrafluorophenylene (**61**) [49] or octafluoro-9,10-anthracenylene (**65**) [50] linkers. The synthesis started with a double Pd-catalyzed Sonogashira cross-coupling reaction of phenylacetylene **50** and 1,4-dibromotetrafluorobenzene. The resulting bis[aryl(ethynyl)]tetrafluorobenzene **59** was able to undergo a 2-fold [4 + 2] cycloaddition reaction with cyclopentadienone **2**, affording polyaromatic **60** in a 70% yield. The final step was the Scholl cyclodehydrogenation of polyphenylene **60** by reaction with DDQ in the presence of TfOH at 0 °C, to afford NG **61** in a good yield. The helical arrangement of these three covalently linked molecular fragments leads to the existence of a chiral axis which gives rise to a racemic mixture. The axis-based helical chiral structures were confirmed by single-crystal X-ray diffractometry and chiral HPLC. The rotational isomerization barrier of C_6_F_4_ ring for the analogue of **62**, with two *tert*-butyl groups substituted by protons was determined as 24.6 kcal/mol at 40 °C with a half-life of *t*_1/2_ = 107 min. Employing the similar synthetic procedure, NG **65** was also synthesized as a chiral axis-based chiral NG by starting with octafluoroanthracen. Recently, An and co-workers reported an atropisomeric chiral nanographene with chirality purely stemming from an axially chiral binaphthyl. As shown in Scheme 7, the Diels–Alder reaction of benzo[*b*]naphtho[2,3-*f*]oxepine **66** with tetrabromothiophene-*S*,*S*-dioxide in toluene followed by oxidative aromatization in the presence of DDQ afforded tetrabrominated aromatics **67** in an 81% yield. Subsequently, fourfold Suzuki–Miyaura cross-coupling of polybrominated compound **67** was performed, affording hexaphenylbenzene **68** in an 85% yield. Then the Scholl cyclodehydrogenation of compound **68** was carried out in DDQ/TfOH to afford NG **69** in a 64% yield. By the selective dimerization of azopine-containing, HBC-monomer **69**, the novel BINOL-like, atropisomeric chiral oxa-nanographene **70** was obtained in high yield. Meanwhile, this NG **70** can also be synthesized from compound **68** through oxidative cyclodehydrognation and dimerization in one pot. The isomerization barrier was determined as over 35 kcal/mol at 170 °C, and 37 kcal/mol by DFT calculation, indicating a high degree of optical stability of pure enantiomers [51].

**Scheme 8:**
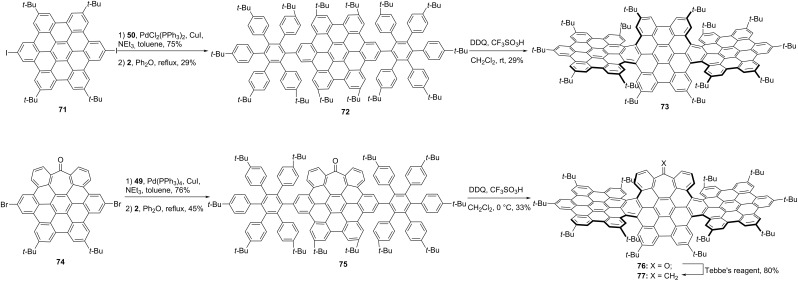
Synthesis of “HBC-trimers”-based nanoribbons.

When three HBCs were fused together linearly, the graphene nanoribbons would be constructed. Wang [46] and Campaña [52] groups separately reported the synthesis of graphene nanoribbon **73** (Scheme 8), which contains four [5]carbohelicenes due to bulky *tert-*butyl groups as lateral chains. In brief, diiodide NG **71** reacted with phenylacetylene **50** through Sonogashira cross-coupling, followed by Diels–Alder reaction with tetracyclone **2** to afford precursor **72** in an overall 22% yield. Then the final helical NG **73**, which contains different conformations was obtained through Scholl reaction in the presence of DDQ and TfOH in a 29% yield. In careful checking of conformations of each fraction, except for achiral *meso* isomers (*P*,*P*,*M*,*M*), a pair of helical enantiomers were confirmed (*M*,*M*,*M*,*M* and *P*,*P*,*P*,*P*). Whereas, the nanoribbons **76** and **77**, which contain a seven-membered ring in the molecular skeleton, following the similar synthetic procedure from the dibromo **74** only gave achiral *meso* isomers (*P*,*P*,*M*,*M*) [52].

**Scheme 9:**
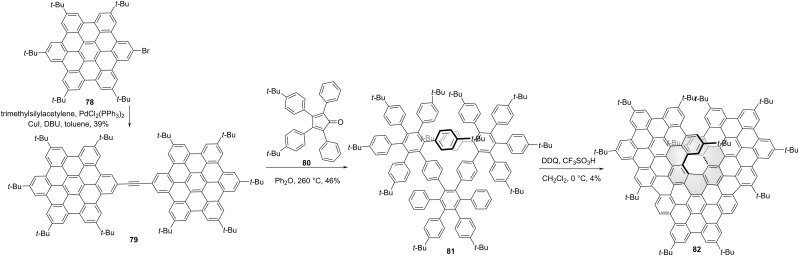
Synthesis of “HBC-trimers”-based, triangle-shaped chiral nanographenes.

**Scheme 10:**
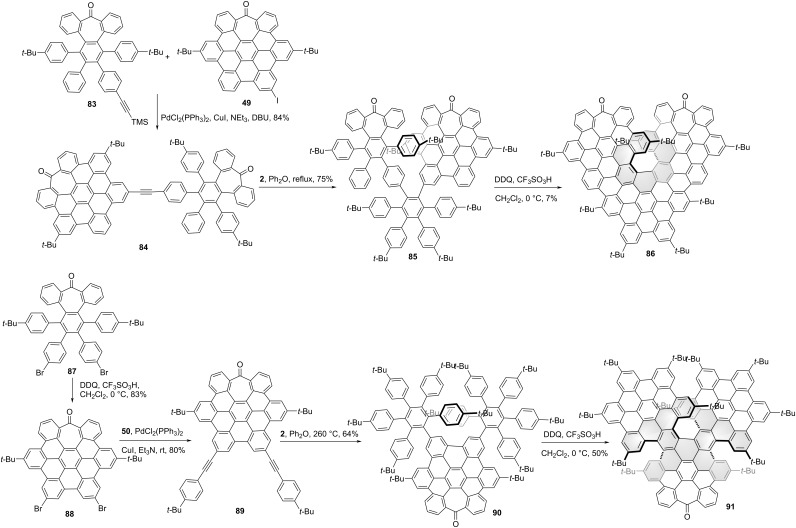
Synthesis of “HBC-trimers”-based, triangle-shaped chiral nanographenes.

Wang and co-workers synthesized the triangular NG **82** by oxidative cyclodehydrogenation of the hexaphenylbenzene-trimer **81** [53]. As shown in Scheme 9, two-fold Sonogashira cross-coupling reaction of trimethylsilylacetylene and monobromo **78** afforded HBD-dimer **79**. Then the Diels–Alder reaction of compound **79** and tetracyclone **80** gave compound **81** in a medium yield. Due to the crowdedness of adjacent HBC units, a chiral [7]helicene **82** was formed during the Scholl reaction of precursor **81**. Campaña and co-workers synthesized a [7]carbohelicene-centered HBC-trimer **86** with two tropones [54] in the HBC units. As shown in Scheme 10, Sonogashira cross-coupling reaction of alkyne **83** and iodide **49** afforded compound **84** in a high yield. Then the precursor **85** was obtained by Diels–Alder reaction of **84** and tetracyclone **2**. The final Scholl cyclodehydrogenation of **85** afforded the HBC-trimer **86**. The non-linear optics of NG **86** and the chiroptical properties of enantiomers were well studied. The CPL spectra centered at 610 nm were observed for both enantiomeric forms and the *g*_lum_ was determined as 2 × 10^−3^. Due to large racemization barrier for [7]helicene, neither racemization nor decomposition was observed for optical pure **86** at 180 or 200 °C for 24 or 4 hours. Recently, they reported another HBC-trimer **91** consisting of only one tropone [55]. Dibromo **87** was converted into its corresponding distorted *hept*-HBC **88** by oxidative cyclodehydrogenation in the presence of DDQ/TfOH in an 83% yield. Through two-fold Sonogashira coupling reaction with 4-*tert*-butylphenylacetylene (**50**), compound **88** was converted to alkyne **89**. Then compound **89** was subjected to a double Diels–Alder reaction with cyclopentadienone **2**, affording precursor **90** in a 64% yield. By the steric effect of the existing *tert*-butyl groups in precursor **90**, another two extended carbo[5]helicenes were formed in the final Scholl reaction of precursor **90** except for the centred carbo[7]helicene in the final structure **91**. Initial analysis of the final products by chiral stationary phase HPLC suggested four main peaks were detected, belonging to two pairs of enantiomers. Notably, one pair of enantiomers can transform to the thermodynamically more stable (*M*,*M*,*P*)-**91** and (*P*,*P*,*M*)-**91** enantiomers after 16 hours heating. Compared to structures **82** and **86**, the photoluminescence quantum yields of **91** was significantly improved. The enantiomers of **91** were proved as NIR-CPL emitters with 600 to 800 nm range of emission and g_lum_ values of 3 × 10^−3^ both in solution and in the solid state.

**Scheme 11:**
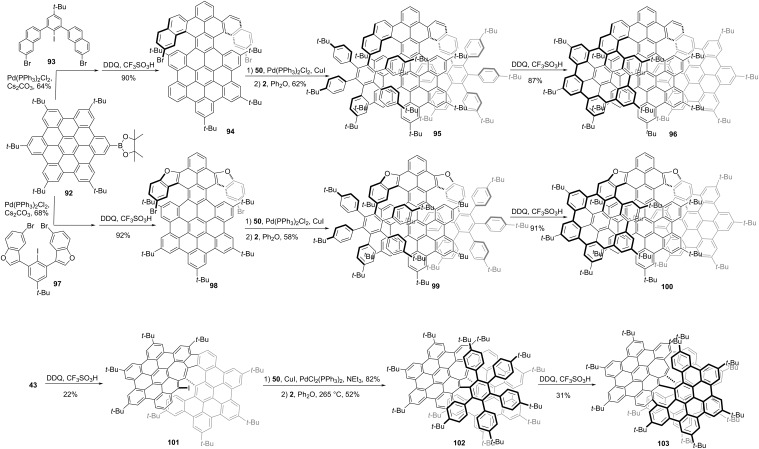
Synthesis of HBC-based multilayer nanographenes.

Multilayer (e.g., bilayer and trilayer) graphenes that are composed of stacked graphenic layers exhibit outstanding electronic and optical features which are not available with single-layer graphenes. The helical bilayer NG **58** developed by Martín and co-workers has been described. Recently, some more complex multilayer NGs were reported. As shown in Scheme 11, Tan and co-workers reported trilayer chiral nanographenes **96** and **100** using helicene or oxa-helicene as the linkers, respectively [56]. The first π-extension started from the borylated penta-*tert*-butyl HBC **92**. The chemical selective Suzuki−Miyaura cross-coupling reaction between **92** and **93** followed by Scholl oxidation produced compound **94** in an overall 50% yield. Alkyne **50** was coupled to **94** by Sonogashira coupling (Scheme 11), followed by Diels–Alder reaction with tetracyclone **2**, giving the phenylethynyl-functionalized double-helical nanographene **95**. The conversion of **95** into nanographene sheets by Scholl oxidation finally afforded the trilayer nanographene **96**. And the trilayer nanographene **100** containing two oxa[6]helicenes was obtained through a synthetic route similar to that of **96**. The structure of hydrocarbon NG **96** exhibited a typical overlapped structure with an interlayer distance of 4.1 Å and consisted of two [8]helicenes as the linker between two HBC layers suggested by DFT optimized structure. The furan containing NG **100** revealed a looser conformation than compound **96** with less overlap between two layers and a longer interlayer distance of 4.9 Å suggested by single crystal X-ray diffraction. The enantiomers of (*P*,*P*)-**96** and (*M*,*M*)-**96** or (*P*,*P*)-**100** and (*M*,*M*)-**100** were well separated by chiral HPLC, and the CD and CPL spectra were investigated for each enantiopure compound. The g_lum_ values were measured as 1 × 10^−3^ and 3 × 10^−3^ for **96** and **100**, respectively. Following their reported structure **44**, Feng, Ma, and co-workers reported a helical bilayer, non-benzenoid nanographene **58** which contains a [10]helicene with two embedded heptagons as a novel chiral moiety (Scheme 11). By using the same intermediate **43**, the iodine-NG **101** together with NG **44** was obtained in a 22% yield. Then compound **101** was coupled with 4-*tert*-butylphenylacetylene through a Sonogashira reaction followed by Diels–Alder reaction with tetracyclone **2**, to give the precursor **102** in an overall 40% yield. The Scholl reaction of **102** using DDQ/TfOH at 0 °C provided the NG **103**. Single-crystal X-ray diffraction analysis of **103** clearly elucidates a closer interlayer distance of 3.2 Å compared with **96** and **100** [57]. The resolution of racemic **58** into its two enantiomers was achieved by chiral stationary phase HPLC. The CPL spectra showed a maximum centered at 688 nm with *g*_lum_ as 1.3 × 10^−3^.

### Chiral NGs containing more than three HBC units

**Scheme 12:**
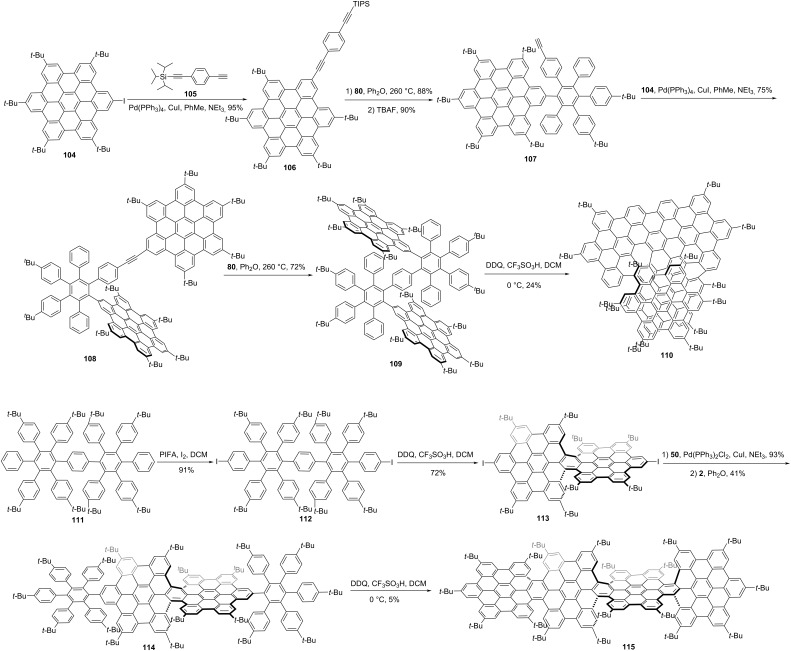
Synthesis of a chiral nanographene constructed by “HBC-tetramers”.

Recently, by fusing four HBC units together, Gong and co-workers synthesized a novel chiral NG **110** (Scheme 12) with a pentadecabenzo[9]helicene core fragment [58]. The synthesis of **110** started from iodohexabenzocoronene **104**. A Sonogashira coupling between **104** and compound **105** gave compound **106**. Then selective Diels–Alder reaction between compound **106** and tetracyclone **80**, followed by removing the triisopropylsilane protective group using tetrabutylammonium fluoride (TBAF) provided compound **107**. Then, a second Sonogashira coupling between **107** and **104** produced compound **108**, followed by a second Diels–Alder reaction between **108** and **80**, which formed intermediate **109** in a high yield. Finally, DDQ and TfOH induced the Scholl oxidation of **109** to generate **110** in a yield of 24%. Single-crystal X-ray diffraction unambiguously confirmed the helical conformation and a pair of enantiomers. Chiral resolution of enantiomers was achieved by chiral HPLC. Due to the large conjugated structure, NG **110** exhibited a significantly red-shifted emission in the near-infrared (NIR) region. Furthermore, the structure extension causes significant improvements both in the fluorescence quantum yield and CPL properties. The dissymmetry factors g_lum_ of (*P*)- and (*M*)-**110** at 684 nm were up to 4.50 × 10^−2^ and 4.22 × 10^−2^, respectively. Wang and co-workers reported a chiral graphene nanoribbon **115** (Scheme 12) by linearly fusing four HBS units in a helical manner [46]. The synthesis started by construction of a HBC-dimer **111**. *para*-Iodization of **111** gave compound **112** in a 91% yield. Scholl oxidation of **112**, and then Sonogashira coupling of **113** yielded the bisalkyne, which was ready for a second Diels−Alder reaction to give precursor **114**. Through dehydrocyclization induced by DDQ and TfOH, precursor **114** was transformed to nanoribbon **115** in a 5% yield. This nanoribbon is 4.3 nm in length, with an end-to-end twist of 117° as indicated by single-crystal X-ray diffraction. Except for the helical conformation, two other isomers, in the waggling or mixed configuration were also generated in the last oxidative cyclodehydrogenation step, and they could be separated via preparative thin-layer chromatography. The *P*- and *M*-isomers of the helical conformation of **115** were separated by chiral HPLC, and the thermal racemization barrier was determined as 44.7 kcal/mol at 260 °C.

**Scheme 13:**
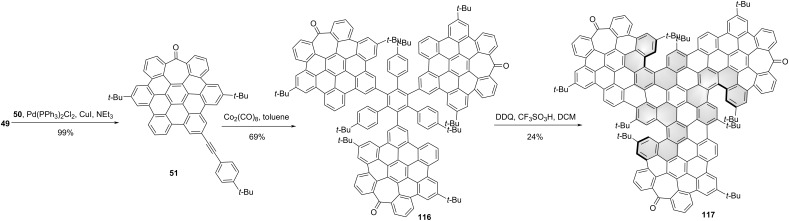
Synthesis of a triskelion-shaped nanographene constructed by four HBCs.

Campaña and co-workers also reported tropone-containing, four HBC-fused NG **117** [59]. The synthesis started with the preparation of the distorted HBC analogue **49**, bearing an aryl iodide for the subsequent Sonogashira cross-coupling reaction with alkyne **50** to give **51**. The precursor **116** containing three pre-existing HBCs was synthesized through Co-catalyzed cyclotrimerization of compound **51**. Finally, compound **116** was subjected to a final oxidative cyclodehydrogenation to create the central HBC unit, leading to NG **117** (Scheme 13) Three carbo[5]helicene moieties were formed during the final Scholl reaction due to the incorporation of bulky *t-*Bu substituents. For the final NG **117**, two diastereomers, *C*_3_-symmetric (*P*,*P*,*P*/*M*,*M*,*M*)-**117** and *C*_1_-asymmetric (*P*,*P*,*M*/*M*,*M*,*P*)-**117** were separated and each racemic diastereomer was resolved into the enantiomers by chiral HPLC. The CPL spectra of both enantiomers show a maximum centered at 643 nm, a *g*_lum_ value estimated as 3 × 10^−4^ for (*P*,*P*,*P*/*M*,*M*,*M*)-**117**. The g_lum_ of (*P*,*P*,*M*/*M*,*M*,*P*)-**117** was measured as 2 × 10^−4^.

**Scheme 14:**
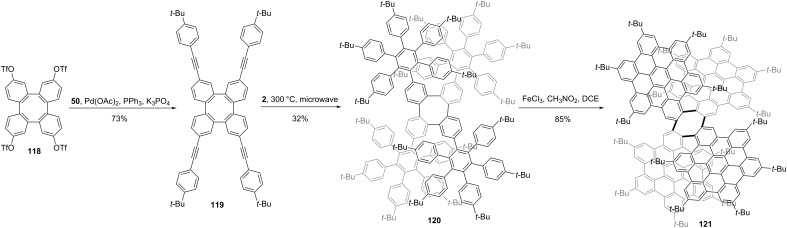
Synthesis of a three-dimensional nanographene bearing four HBCs.

Cyclooctatetraphenylene (COT-Ph) is a π-conjugated scaffold, whose three-dimensional (3D) geometry is based on its saddle shape. Martín and co-workers reported the 3D NG **121** by the introduction of four HBC units into COT-Ph [60]. As shown in Scheme 14, functionalized COT-Ph reacted with compound **50** through Sonogashira cross-coupling reaction affording compound **119** in a 73% yield. Then Diels–Alder reaction between **119** and **2** gave structure **120** as NG precursor. Compound **120** underwent intramolecular cyclodehydrogenation mediated by FeCl_3_, affording the final NG **121** in a high yield. Due to the two conformations of cyclooctatetraene saddles, NG **121** is a chiral structure and optically pure enantiomers were separated using chiral high-performance liquid chromatography (HPLC), which featured a perfect mirror-image circular dichroism (CD) for both enantiomers.

**Scheme 15:**
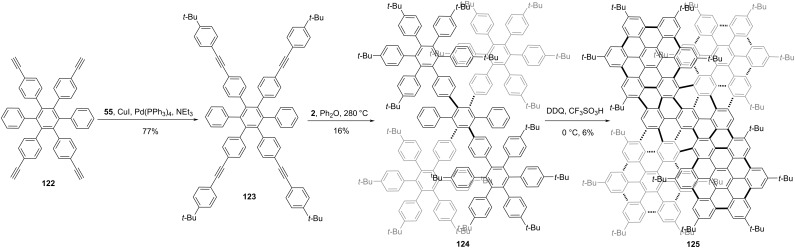
Synthesis of a chiral nanographene constructed by five HBC units.

Wang and co-workers constructed double π-extended undecabenzo-[7]helicene **125**, which composed of five HBC units and 186 sp^2^ carbon atoms [53]. As shown in Scheme 15, a hexaphenylbenzene with four alkyne substitutions was constructed initially. A Sonogashira coupling from **122** to **123**, followed by a Diels–Alder reaction, yielded the polyphenylene precursor **124** in an overall 12% yield. At last, a Scholl oxidation, mediated by DDQ and TfOH, gave the target NG **125** in a 6% yield. Due to the extremely large conjugated structure, compound **125** shows an extraordinary panchromatic light absorption capability (ε = 844 000 M^−1^ cm^−1^ at 573 nm). A pair of enantiomers, (*M*,*M*)- and (*P*,*P*)-configuration was revealed by single crystal X-ray diffraction and optically pure samples of **125** were isolated by chiral HPLC. Meanwhile, a record high electronic circular dichroism (ECD) signal in the visible spectral range (Δε = 1375 M^−1^ cm^−1^ at 573 nm at 430 nm) for enantiopure **125** was obtained.

**Scheme 16:**
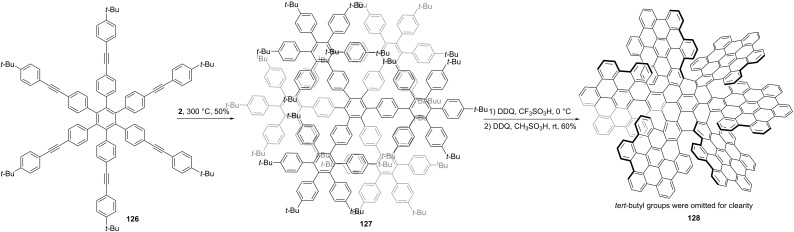
Synthesis of a chiral nanographene constructed by seven HBC units.

In 2019, Wang and co-workers reported the largest atomically precise three-dimensional conjugated chiral nanographene propeller **128**, representing the largest three-dimensional conjugated polyaromatics everprepared using scalable solution chemistry [61]. As shown in Scheme 16, similar to **125**, a hexaphenylbenzene **126** with six alkyne substitutions was prepared first. Through a six-fold Diels–Alder reaction with **2**, compound **126** was converted to **127** in a 50% yield. Finally, NG **128** was synthesized from polybenzenes **127** through a stepwise Scholl reaction from 0 °C to room temperature by removing 84 hydrogens total. NG **128** is composed of seven HBCs and exhibit an extraordinary size, which reaches 3.50 nm in width and 1.25 nm in height. Single crystals X-ray diffraction indicates only one form (*M* or *P*) of enantiomer which can be separated by chiral HPLC. Optically pure enantiomers show a series of strong mirror-image Cotton effects across the whole UV−NIR spectral window.

## Conclusion

As demonstrated by the aforementioned success in synthesizing numerous chiral nanographenes, the HBC is a well-established reference structure to exploit three-dimensional aesthetic structures. The continuously developed novel structures contented our interests and curiosity. To further tune the optoelectronic or photophysical properties of nanographenes for real applications, heteroatom doping was found to be an effective strategy. The introduction of different main group elements beyond nitrogen summarized above would provide more diverse structures and properties. Meanwhile, the methodologies for further functionalization of the existing nanographene molecules are still needed for application purposes.
